# Psychological Distress, Somatic Complaints, and Their Relation to Negative Psychosocial Factors in a Sample of Swedish High School Students

**DOI:** 10.3389/fpubh.2021.669958

**Published:** 2021-07-19

**Authors:** Nóra Kerekes, Btissame Zouini, Sofia Tingberg, Soly Erlandsson

**Affiliations:** ^1^Department of Health Sciences, University West, Trollhättan, Sweden; ^2^Department of Biology, Faculty of Sciences, Abdelmalek Essaadi University, Tetouan, Morocco; ^3^Department of Social and Behavioural Studies, University West, Trollhättan, Sweden

**Keywords:** adolescence, Brief Symptom Inventory, gender difference, psychological distress, somatic health, the experience of physical or psychological abuse

## Abstract

**Background:** Adolescence is a period in life characterized by major neurobiological, physiological, and psychological changes. Those changes may give rise to worsened mental health and an increased prevalence of somatic complaints combined with a negative psychosocial environment. Rapid changes in society, which may also affect young people in several ways, call for a renewed screening of today's adolescents' mental and somatic well-being.

**Aim:** The present study's primary aim was to measure the level of self-rated psychological distress and the prevalence of somatic complaints in a sample of Swedish high school students. As a secondary aim, it identifies gender-specific patterns and examines mental and somatic health in relation to negative psychosocial factors (such as parental alcohol use problems or the experience of physical or psychological abuse).

**Method:** Two hundred and eighty-seven Swedish high school students completed a survey including the Brief Symptom Inventory (BSI) and a questionnaire about the presence of defined somatic complaints. In order to examine the relationship between negative psychosocial factors and mental and somatic health, three groups were formed: those reporting (i) parental substance use problems, (ii) previous experience of abuse, (iii) none of these problems.

**Results:** The majority of the Swedish high-school students (>80%) reported no or only a few problems with psychological distress and no or only one somatic complaint. Female students disclosed a significantly higher psychological distress level captured by each BSI domain. The number of somatic complaints was similarly distributed between the genders. The students rarely reported parental substance use problems, but almost 40% of the male and 50% of the female students indicated the experience of physical and/or psychological abuse. Such negative psychosocial circumstances were related to an increased level of anxiety in the male and an increased general level of psychological distress in female students.

**Conclusions:** The study confirmed female students' higher psychological distress level. Gender differences in the type of somatic complaints, but not in the number were detected. The experience of physical and/or psychological abuse was found to significantly worsen psychological distress in students of both genders.

## Introduction

Many different aspects, inherent and contextual, affect young people's psychological and physical health during adolescence. Biological, psycho-developmental, and social changes, that take place almost simultaneously during this developmental period (1) combined with an intensive structural and functional remodeling of the brain (pruning) (2) have an increased impact on adolescents' general well-being. The reconstruction of the brain runs parallel with hormonal changes, which imply an awakening of the sexual desire and, at the same time, the development of cognitive skills (1). Different norms in social groups, as well as norms in society, have, to a different degree, a life-long impact on individuals. Especially for the adolescent individual, it is vital to learn and follow those norms to be accepted by a group or by society. Furthermore, demands might have a strong influence on young peoples' behavior and well-being (3).

Why the changes mentioned above are general, there are also gender-specific patterns in the adolescent developmental phase. Young females usually exhibit an earlier social and cognitive maturation than their age-matched male counterparts (4). Moreover, they also experience a greater deal of psychological distress compared to young males (5) and a greater proportion of them complain about physical health problems (6, 7). Most commonly reported by female adolescents are generalized anxiety, panic syndrome, social anxiety, depression (5, 8, 9), and headaches (7). Compared to young women, however, male adolescents experience more social isolation (10), and their mental problems are not seldom observed through norm divergent behavior (11).

Both female and male adolescents report that all types of supportive relationships which are based on reciprocity, understanding, and respect have a positive effect on their psychological health (12); while negative, psychosocial factors seem to increase the prevalence of both physical and mental health complaints (7, 13). Another important part of adolescents' lives that might impact psychological and physical health is their school environment. Concentration difficulties and low self-esteem, headaches, allergies, and gastrointestinal complaints can result from students' worry and stress experienced in the school environment. Murberg and Bru (14) investigated school-related stress and psychosomatic symptoms in adolescents. They found that although adolescent girls experience more psychosomatic symptoms than their male classmates, peer-related difficulties at school are stronger associated with boys' psychosomatic symptoms than girls. The authors also defined four main dimensions of school-related stress: (i) difficulties with peers at school, (ii) worries about school achievement, (iii) schoolwork pressure, and (iv) conflicts with parents and/or teachers (14). In accordance with those findings, studies focusing on protective or salutogenic factors deriving from the school-environment in adolescents' life suggest that factors promoting well-being are closeness to parents and friends (15). High-quality interpersonal relationships have also been found to promote higher academic achievements (15).

Parents' behavior toward their children during adolescence are essential for their children's well-being (16–18) and can have a long-term effect on their mental health as adults (19). According to Conger et al. (20), a disadvantaged family environment can lead to children showing more negative emotionality, being less agreeable, and lacking conscientiousness. The strength or more clearly the weakness of personality dimensions, like emotional stability, conscientiousness, and agreeableness, can predict the presence of severe psychiatric ill-health in adolescents, for example, self-harm behaviors and suicidal depression (21). Notably, the relationship between personality and mental disorders is not affected by gender or age when a disorder was diagnosed (22). And again, besides family and school environment, societal norms, and cultural input during the developmental period of adolescence have a remarkable impact on adolescents' health and well-being (12, 23).

While relations of a destructive character can lead to mental distress (12) and may also be coupled to an increased prevalence of physical complaints (24), the importance of supportive, good relationships for physical and psychological health cannot be underestimated. One crucial component of well-being during adolescence is to search and find meaning in life. Previous research has shown a possible link between the lack of meaning in life and the prevalence of psychosomatic symptoms in youth (25). Performed studies over several years across countries and cultures have shown that meaningfulness (sense of coherence) can be a powerful coping strategy and a potential protective factor in stressful situations (26).

The economic crisis during 1990 led to an intensified debate in Sweden concerning the young generation and their psychological health. Recommendations from an expert panel at the Royal Academy of Science to investigate and find the key issues behind mental ill-health among young people did not straighten out the question marks. Over the period of the deep economic recession in 2007 and 2008, actually a strong social net and support together with in-depth relief programmes in Sweden could compensate for the, in many other countries seen, worsened mental health (27–29). Recent longitudinal, interdisciplinary research studies have pointed to economic vulnerability during the 1990s and school stress during the last couple of decades as explanations to risk factors for the elevation of psychosomatic symptoms in young people (30).

Based on the findings from interdisciplinary research, a holistic approach, by which both somatic and mental health in a complex bio-psycho-social matrix are in focus, were found to be relevant and sought-after aspects of adolescent health research. To meet some of these goals, in the present study the prevalence and gender-specific distribution of self-reported somatic and mental health were investigated in a sample of Swedish high school students. The sample originated from an urban area, with an average Swedish socioeconomic status, on the West Coast of Sweden the year of 2018. Our aim with the present investigation is to contribute to the research area by searching for several underlying aggravating circumstances that might lead to adolescent health problems. The results presented may also open possibilities to compare perceived threats and problem areas on different levels in the youths' life, as well as effects of cultural and societal norms on their health.

## Method

The “Mental and Somatic Health without borders” (MeSHe) project (31) is an international project focusing on culture-specific risk- and protective factors of adolescent substance use and aggressive antisocial behavior. This project collects data by a standardized survey (the MeSHe survey) that includes seven validated scales (Life History of Aggression; Brief Symptom Inventory; Alcohol Use Disorder Identification Test; Drug Use Disorder Identification Test; Positive Affect and Negative Affect Schedule; Godin Leisure-Time Exercise Questionnaire and a personality inventory). The survey also includes a health questionnaire assessing the respondent's age, gender, nationality, and defined negative psychosocial factors and somatic health.

The present study's data collection was performed at a high school in Western Sweden during a week in May 2018. Teachers were present in the entire survey, but students could work in privacy alone. The time devoted to the students to complete the questionnaires was 60 min. In the meantime, students who chose not to participate in the survey worked with an alternative task. Students who required extra time or support to fill in the questionnaire were offered help, which implied that those with reading/writing difficulties could be included in the survey. The students placed the completed survey in a separate, sealed envelope and gave it to the teacher.

### Study Population

The study population consisted of high school students in a medium-large city in the West part of Sweden. The inclusion criteria for participation were to be able to read and understand Swedish. The principal at the high school asked 407 students (17% studied at the Program of Business and Administration, 17% at the Program of Child and Recreation, and 66% at the Program of Business Management and Economics) about their interest in participating in the study. Of those who were asked to participate, 29.7% (121 students) chose not to participate in the study (attrition rate), which resulted in a response rate of 70.3% (286 students). Of 286 responding students, 46 (16%) did not fully complete the BSI questionnaires (internal dropout).

Moreover, one participant declared “other gender,” and four did not answer the question about their gender, adding a proportion of 1.75% of the data's internal dropout in the gender-specific analyzes. Thus, the final study population of 281 high school students (114 male and 167 female) were included in the gender-specific psychological distress analyses. The youngest participant in the study was 15 years old, the oldest 20 years old, and the average age of the study population was 17.30 years, with a standard deviation of 0.60 years.

The number of missing answers varied in the somatic health questionnaire section between zero/no missing answer (regarding complaints about constipation for a more extended period than 14 days) and nine (regarding previous head injuries), which resulted in a response frequency between 286 and 277 students. Due to four students who could not be categorized either as male or female gender, the gender-specific analyses of the prevalence of somatic complaints were based on answers from 163 to 164 females and 109 to 114 male students.

### Instruments

#### Brief Symptom Inventory (BSI)

BSI is a self-assessment form that measures the individual's perceived mental distress (32). It consists of 53 items divided into nine domains (primary symptoms scales) and can be described with the full scale of the General Severity Index (GSI). Each question measures to what extent individuals estimate their experiences according to the claims of the question on a Likert scale ranging from zero to four (0 = not at all, 1 = a little, 2 = moderate, 3 = pretty much, 4 = very much) (32). The BSI instrument has been tested for reliability in its original English form and in the Swedish translation for an adult population (33, 34). The instrument was previously used in an adolescent population in a Turkish (35) and an Arabic (13) translated version. In the current study, the high school students received a Swedish translation of the original English version of BSI, and asked to estimate their mental suffering during the past year. Data analyses confirmed that the inventory and its nine domains had acceptable internal reliability in our study population (Cronbach α = 0.97 for GSI). The following text describes how the nine domains, including their internal reliability, capture psychological distress.

##### Somatization

Mental suffering can manifest itself in physical symptoms, such as cardiovascular, digestive and respiratory system or other areas affected by the autonomic nervous system (32). Examples of items are: Faintness or dizziness; Pain in the heart or chest; Nausea or upset stomach. Internal reliability of the Somatization domain was α = 0.78.

##### Obsessive Compulsive Behavior

Characteristic signs of obsessive compulsive behavior are recurrent and irresistible thoughts and actions such as repeatedly double-check if a task is performed, concentration difficulties, difficulties making decisions (32). Examples of items are: Trouble remembering things; Feeling blocked in getting things done; Having to check and double-check what you do. The internal reliability of this domain was α = 0.84.

##### Interpersonal Sensitivity or Social Insecurity

Social insecurity is considered to have a low intrinsic value, feelings of concern, being highly uncomfortable in social interactions (32). Examples of items are: Feeling that people are unfriendly or dislike you; Feeling inferior to others; Feeling self-conscious with others: The internal reliability of the domain was α = 0.85.

##### Depression

A broad repertoire of symptoms indicate a state of depression, including dysfunctional effects, decreased interest in things that have been of interest before, low energy levels, and a sense of hopelessness (32). Examples of items are: Thoughts of ending your life; Feeling lonely; Feeling blue; Feeling no interest in things. The internal reliability of the domain was α = 0.87.

##### Anxiety

This domain is characterized by typical symptoms of severe anxiety, such as unprovoked anxiety, panic attacks, muscle tension, restlessness, and nervousness (32). Examples of questions are: Nervousness or shakiness inside; Suddenly scared for no reason; Spells of terror or panic. Internal reliability in the domain was α = 0.84.

##### Hostility

The hostility domain is characterized by threatening behavior occurring in thought, emotion, and action. Standard features are to become easily irritated, get into trouble at a fast rate, feeling an urge to break something, and outbursts of anger (32). Examples of questions are: Feeling easily annoyed or irritated; Temper outbursts that you cannot control; Feeling an urge to beat, injure, or to harm someone. Internal reliability in the domain was α = 0.82.

##### Social Phobia (i.e., Phobic Anxiety)

Social phobia has a resemblance to agoraphobia, meaning that the person feels uneasy staying in a large human gathering, when using collective transport, or be in public places (32). Examples of questions are: Feeling afraid in open spaces; Feeling afraid to travel on buses, subways or trains; Having to avoid places or activities experienced as frightening. Internal reliability in the domain was α = 0.84.

##### Paranoid Ideation

The paranoid mindset is assumed to be a natural syndrome experienced as symptoms with adverse effects, such as projection, hostility, distrust, self-centering, and suspicion that someone will deprive you of your autonomy (32). Examples of questions are: Feeling that others are to blame for most of your troubles; that most people cannot be trusted; that you are watched or talked about by others. Internal reliability in the domain was α = 0.81.

##### Psychoticism

This domain covers the area between a deviant lifestyle and total psychosis. Measured here is however, a non-clinical population's socially, abnormal behavior (32). Examples of questions are: The idea that someone else can control your thoughts; Feeling lonely even when you are with people; The idea that you should be punished for your sins; Never feeling close to another person; The idea that something is wrong with your mind. Internal reliability in the domain was α = 0.80.

#### The Somatic Health Questionnaire

A part of the MeSHe survey, is a measure of the presence of selected physical complaints and diseases. The questionnaire was developed by the Swedish project leader (NK) and based on a similar questionnaire in a nation-wide twin study in Sweden (36). The MeSHe Somatic health questionnaire measures the presence of the following, selected physical complaints or diseases: Problems with diarrhea or constipation for a period longer than 14 days; the existence of head injury; cancer/leukemia or other tumor diagnoses; epilepsy; rheumatological diseases; diabetes, asthma; other allergies; skin diseases; celiac diseases; tuberculosis; migraines; thyroid diseases. The MeSHe Somatic health questionnaire previously showed high test-retest reliability (7).

The presence of *negative psychosocial factors* in the students' life was measured with four questions: (i) “Do any of the adults you live with have a problem with alcohol (alcoholism)”? (ii) “Do any of the adults you live with have a problem with drugs?” (iii) “Have you ever experienced physical abuse (for example, have you been pushed, kicked, beaten, slapped, etc.)”? and (iv) “Have you ever experienced psychological abuse (for example, have you been threatened, forced to do something that feels wrong, violated by humiliating and insulting words, etc.)”? Three groups were formed based on the affirmative answers:

- “Parental Substance use Problems” (PSP), i.e., those indicating having adults in their life with alcohol and/or drug use problems.
- “Physical or Psychological Abuse” (PPA), i.e., those indicating having experienced physical and/or psychological abuse.
- The comparison group (CG), i.e., those dissenting to all four questions regarding negative psychosocial factors.

### Data Analysis

The computer program Statistical Package for the Social Sciences (SPSS) software version 24.0 (IBM) was used for data analysis. Since data differed significantly from the normal distribution (*p* < 0.001 in Shapiro-Wilk test), and the calculated scores were not normally distributed (Kolmogorov-Smirnov test' significance <0.05), non-parametric statistical analyses were performed. These were the Mann-Whitney *U*-test for comparing the scores of male and female students, and the Kruskal-Wallis *H*-test for comparing the mean ranks between adolescents belonging to the different groups (CG, PSP, and PPA), followed by a *post-hoc* test for analyzing pairwise differential interactions between the three groups. All the above analyses were two-tailed, and the significance level was set at 5%.

Concerning the somatic health dimension, contingency square analysis was performed to assess the relationship between somatic symptoms and diseases, on the one hand, and psychosocial variable groups, on the other. The strength of the statistically, significant relationship was evaluated using Cramer's V effect size [values from 0.07 to 0.20 indicate a small effect, 0.21 to 0.35 a medium effect, and 0.36 and above suggesting a large effect (37)], or Eta^2^ = Z^2^/(n − 1) [0.01−0.05 indicates a small effect size, 0.06 to 0.13 a medium and values above 0.14 a large effect size (38)]. Corrections for Type I errors were performed by Bonferroni correction (39, 40) setting the significance cut-off at α/n (0.05/3 = 0.017), where n refers to the number of compared groups.

### Ethical Consideration

The Regional Ethical Review Board in Gothenburg approved the study; protocol No. 689 - 17. Ethical approval ensures that the study follows the Helsinki Declaration's and the Swedish law with ethical guidelines for scientific research on people. The Act on ethical review of research involving humans according to the Swedish Law (2003: 460) § 18 declares: “If the research person has reached the age of 15 but not 18 and realizes what the research means for him or her, he or she shall be informed of and consent to the research in the manner specified in §§ 16 and 17” (41). The Ethical Review Board required all high school students to be informed of available support organizations when the assessments took place. All students received written and oral information that participation was voluntary and anonymous. Furthermore, they were informed that participation would not affect their study results, and that they could cancel their participation at any time. The surveys collected were processed so that only authorized persons had access to the data material.

## Results

### Level of Psychological Distress

Almost one third (29.6%) of the students reported no symptoms on the Brief Symptom Inventory (BSI) and about half of them (52.1%) experienced a low level of psychological distress. Moderate problems with distress was reported by 15% of the students, and a very high level of psychological distress by 3.2%. Mirroring the over-all distribution of the level of distress, the mean value of the students' psychological distress, measured with the General Severity Index (GSI), was 0.93 (*SD* = 0.68, median value 0.75). The response rate on BSI's different primary domains varied between 264 and 272. Mean values of the GSI and the nine primary domains in the whole study population and by gender are summarized in Table 1. The mean values of the domains varied between 0.56 (Psychoticism) and 1.29 (Obsessive compulsive behavior). The primary domains of Obsessive compulsive behavior, Anxiety, Paranoid Ideation, and Depression had the highest scores of all. The five most highly rated items on item level were: A feeling of nervousness or shakiness inside (*M* = 2.13); Blocked in getting things done (*M* = 1.85); Difficulty in making decisions (*M* = 1.5); Sensing that most people cannot be trusted (*M* = 1.44); Being easily hurt (*M* = 1.28).

**Table 1:** Self-reported psychiatric problems in the general population of a sample of Swedish adolescents (*N* = 286).

**BSI subscales**	**Total sample *M* (*SD*) min–max**	***n***	**Males *M* (*SD*) min–max**	***n***	**Females *M* (SD) min–max**	***p***	***Eta^**2**^***
Somatization	0.78 (0.7) 0–3	104	0.48 (0.5) 0–3	160	0.98 (0.75) 0–3	<0.001	0.12
Obsessive compulsive behavior	1.30 (0.86) 0–4	106	0.91 (0.73) 0–3	163	1.55 (0.85) 0–4	<0.001	0.13
Psychoticism	0.56 (0.74) 0–4	107	0.32 (0.59) 0–4	164	0.71 (0.8) 0–4	<0.001	0.07
Depression	1.04 (0.94) 0–4	104	0.64 (0.74) 0–4	163	1.30 (0.96) 0–4	<0.001	0.12
Interpersonal sensitivity	0.97 (0.99) 0–4	108	0.48 (0.68) 0–4	162	1.30 (1.04) 0–4	<0.001	0.16
Hostility	0.88 (0.8) 0–4	108	0.73 (0.78) 0–4	164	0.97 (0.81) 0–3	0.012	0.02
Phobic anxiety	0.61 (0.82) 0–4	106	0.22 (0.52) 0–4	162	0.86 (0.87) 0–4	<0.001	0.15
Anxiety	1.11 (0.87) 0–4	102	0.61 (0.58) 0–4	166	1.42 (0.87) 0–4	<0.001	0.21
Paranoid ideation	1.05 (0.89) 0–4	108	0.69 (0.76) 0–3	164	1.28 (0.89) 0–4	<0.001	0.11
GSI	0.93 (0.68) 0–4	92	0.60 (0.55) 0–4	148	1.14 (0.68) 0–3	<0.001	0.14

Results indicated that the female high school students had a significantly higher general level of psychological distress than the male students. In each BSI domain, female students compared to their male classmates, estimated their psychological distress level higher (each of those with large effect size, except Psychoticism with medium effect size, and Hostility with a small effect size). The domains that were found to distinguish the genders by effect size at most were Anxiety (Eta^2^ = 0.21) and Interpersonal sensitivity/social insecurity (Eta^2^ = 0.16).

### The Prevalence of Somatic Complains

**Figure 1:**
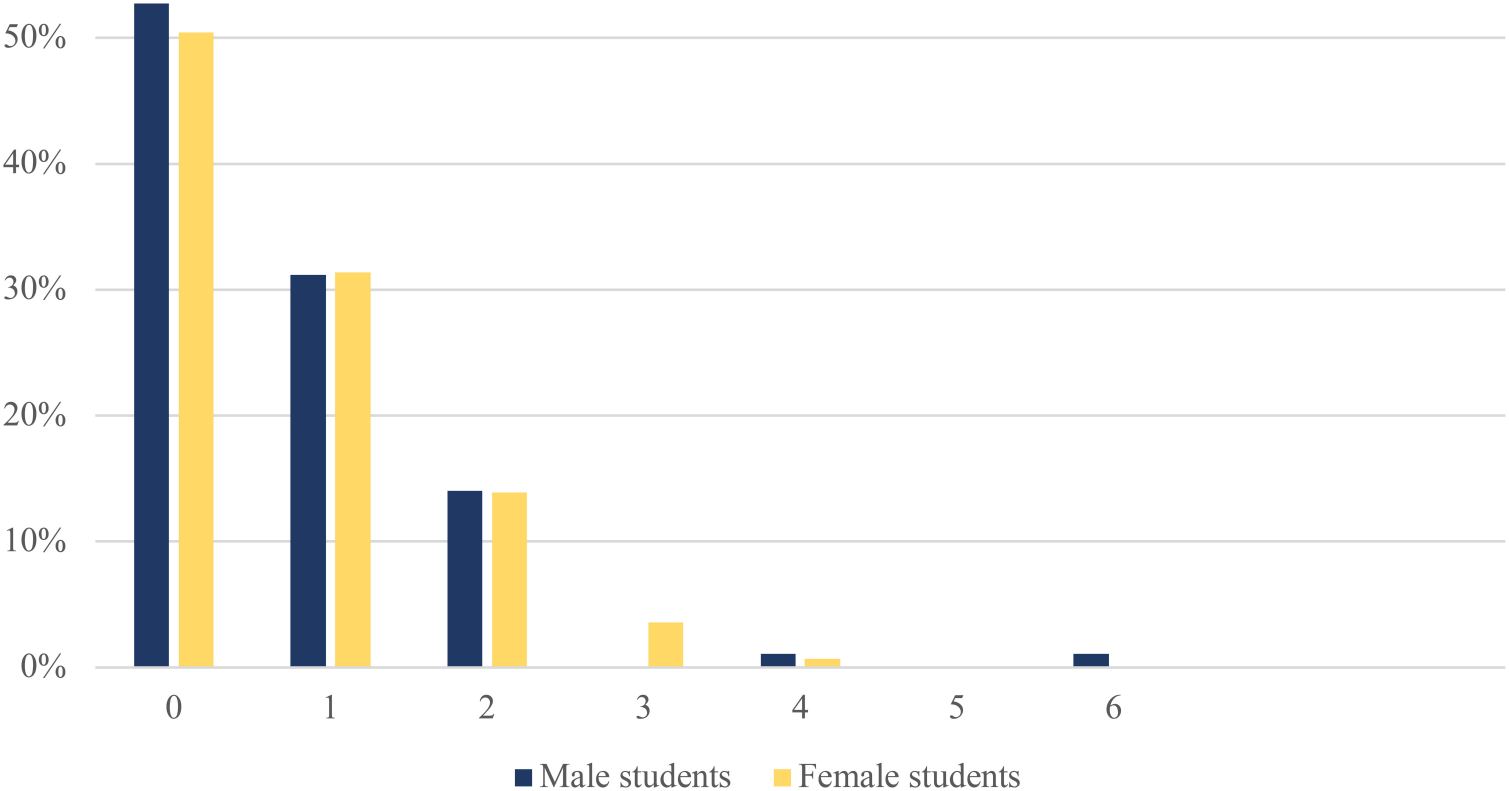
Proportion of adolescents [male (*n* = 93) and female (*n* = 137)] reporting different number of somatic complains.

Half (51.3%) of the students reported no somatic problems at all, another one-third of them (31.3%) indicated having one somatic complaint, and eight students (3.5%) three or more somatic complaints. The prevalence of somatic complaints was similarly distributed among female and male students (Figure 1). The most prevalent somatic complaint was allergy (37.8 and 26.3% in male and female students respectively) and migraine (11.1 and 20.2% in male and female students respectively). Significant differences in the prevalence of somatic complaints between the genders could be found in constipation (1.8% in male and 9.4% in female students; *p* = 0.008) and migraine (11.1% in male and 20.2% in female students; *p* = 0.048). Somatic complaints were significantly more prevalent in female students, while “other allergies” were significantly more prevalent in male students (37.8% in male and 26.3% in female students; *p* = 0.04; Table 2).

**Table 2:** Prevalence of defined somatic symptoms and diseases in a sample of Swedish students (*N* = 286).

	**Total sample (yes/no)^a^ %**	**Males (yes/no)^a^ %**	**Females (yes/no)^a^ %**	**Chi-Square test**		
				***χ^2^***	***p*-value**	**Cramer's V**
Diarrhea	(7/260) 2.6	(4/104) 3.7	(3/156) 1.9	0.83	0.36	0.06
Constipation	(17/253) 6.3	(2/109) 1.8	(15/144) 9.4	6.45	0.008	0.16
Cancer	(1/272) 0.4	(0/110) 0.0	(1/162) 0.6	0.68	0.41	0.05
Epilepsy	(3/274) 1.1	(1/112) 0.9	(2/162) 1.2	0.07	0.79	0.02
Rheumatologic disease	(5/268) 1.8	(2/109) 1.8	(3/159) 1.9	0.01	0.98	0.02
Diabetes	(2/274) 0.7	(1/112) 0.9	(1/162) 0.6	0.07	0.79	0.02
Asthma	(38/235) 13.6	(15/96) 13.5	(23/139) 14.2	0.03	0.87	0.01
Other allergy	(84/187) 31.0	(42/69) 37.8	(42/118) 26.3	4.11	0.040	0.12
Skin disease	(18/251) 6.7	(5/104) 4.6	(13/147) 8.1	1.30	0.25	0.07
Gluten intolerance	(6/268) 2.2	(3/108) 2.7	(3/160) 1.8	0.23	0.63	0.03
Migraine	(45/226) 16.6	(12/96) 11.1	(33/130) 20.2	3.91	0.048	0.12
Thyroid disease	(2/269) 0.7	(1/110) 0.9	(1/159) 0.6	0.07	0.79	0.02

a *n includes those answering yes or no to the question*.

### Distribution of Negative Psychosocial Factor

**Figure 2:**
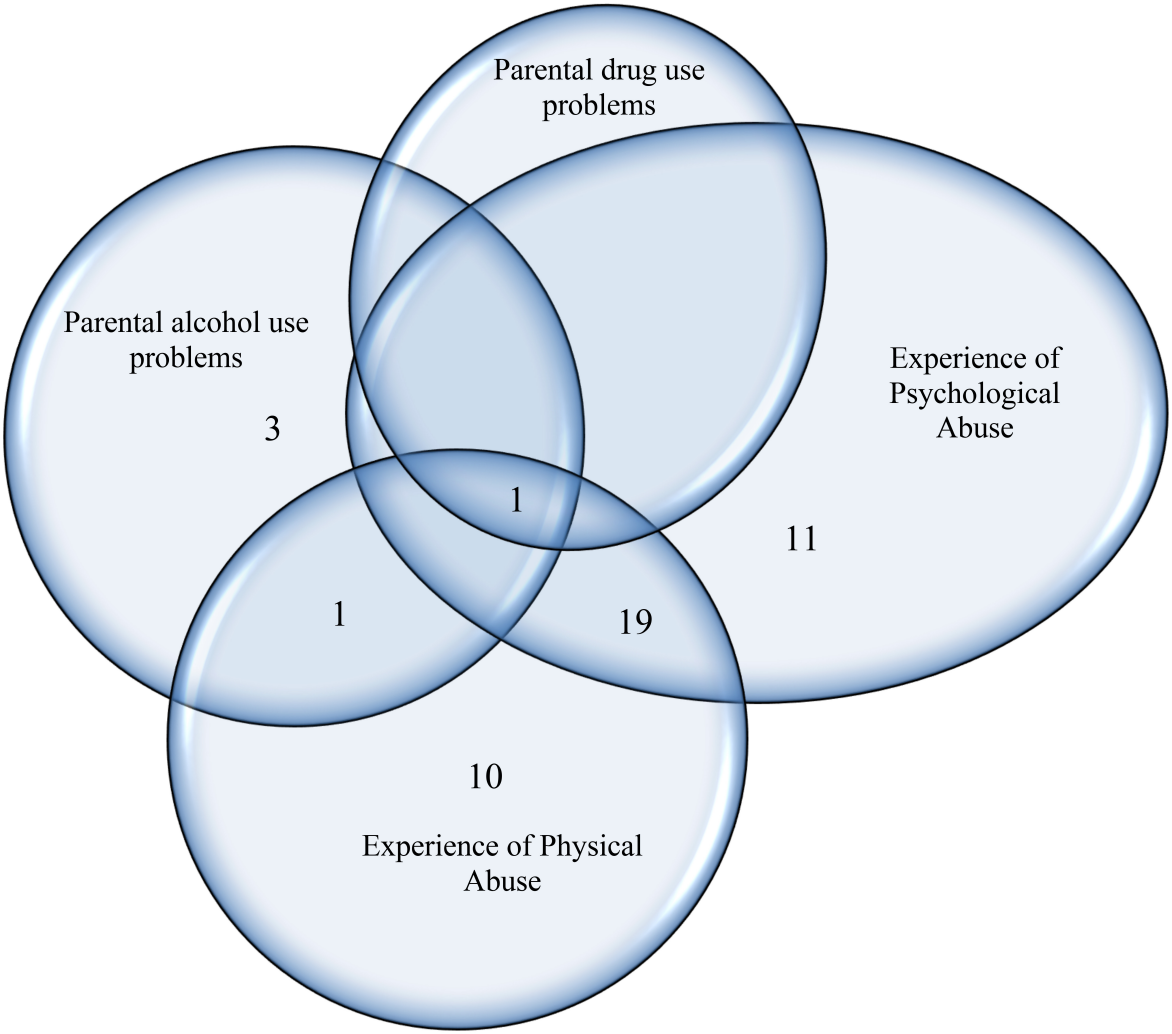
The prevalence of male students reporting the existence of different negative psychosocial problems in a sample of Swedish high school students (*n* = 111).

**Figure 3:**
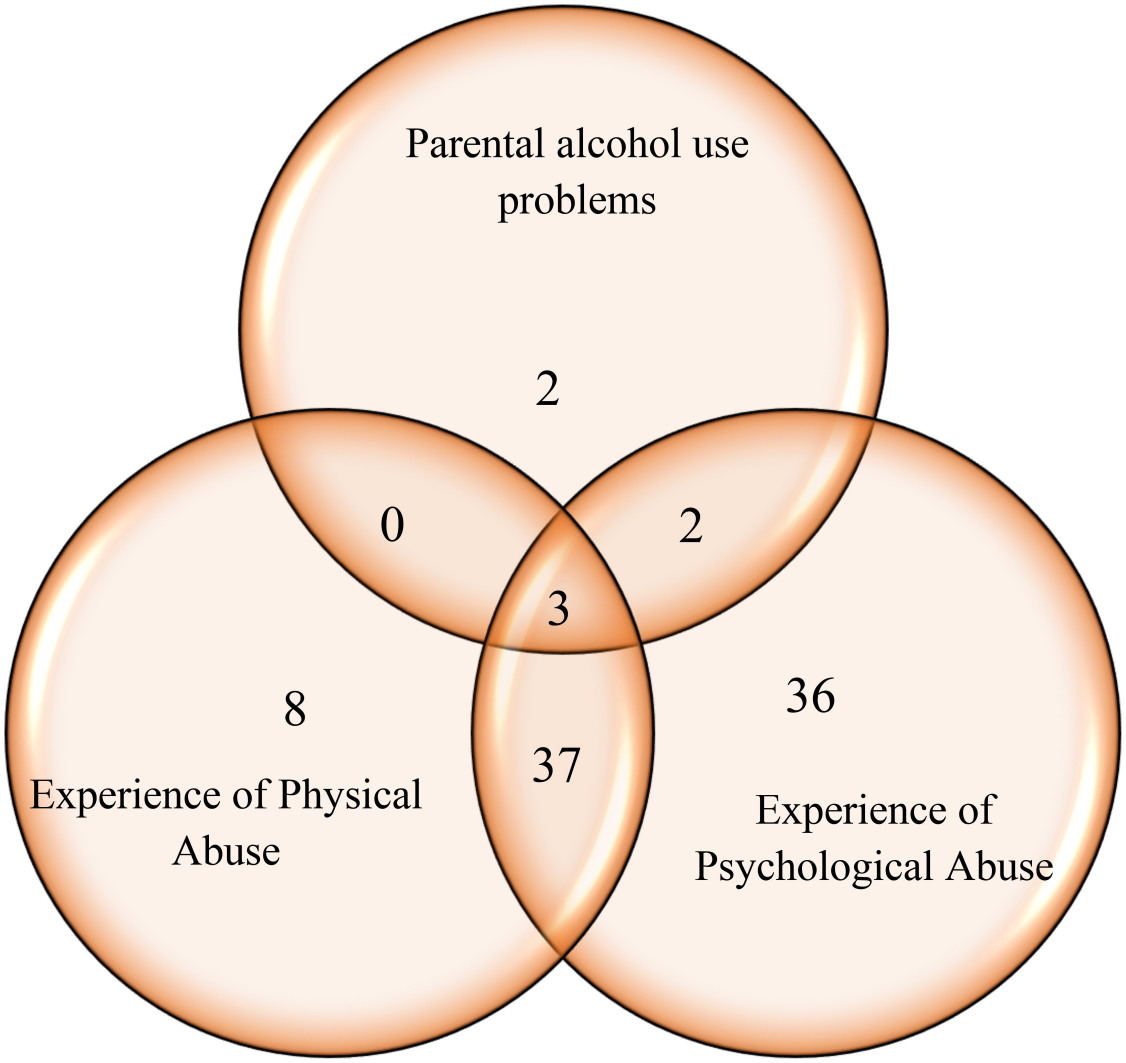
The prevalence of female students reporting the existence of different negative psychosocial problems in a sample of Swedish high school students (*n* = 165).

The overlap of the affirmative answers to the four questions about negative psychosocial factors in the students' life, separated by gender, is illustrated in Figures 2, 3. Only one (1%) of the responding 111 male students reported having adults in his life who had drug problems and four male students (3.6%) were found to have adults with alcohol use problems in their lives. The overlap between parental substance use (drugs or alcohol) and the experience of abuse was reported by two male students (1.8%). Forty two (37.8%) male students reported that they had experienced physical and/or psychological abuse; almost half of them (*n* = 20) reported both physical and psychological abuse (Figure 2).

None of the 165 responding female participants reported having adults with drug use problems in her life. Out of seven females (4%) reporting adults with alcohol use problems in their family, five had experienced abuse (Figure 3). Almost half (47%; *n* = 78) of the female students had experiences of psychological abuse, and nearly one third (29%; *n* = 48) experiences of physical abuse (Figure 3). Of female students who had been exposed to abuse 40 (46.5%) reported the experience of both psychological and physical abuse.

As students reporting both PSP and PPA were very few (two males and five females), no statistical analyses for this group were specifically performed; instead, their reports were accounted for in both the PSP and the PPA groups.

### Psychological Distress and Negative Psychosocial Factor

The presence of an adult(s) with substance use problem (PSP) in the students' life and the experience of physical and/or psychological abuse (PPA) contributed to an increased level of psychological distress in all primary dimensions as well as in the overall level of psychological distress (Table 3). A significant difference could be measured in the GSI between the PSP and the CG groups. At the primary domain level, a significant difference between these two groups was found in Paranoid ideation, Anxiety, Phobic Anxiety, Depression, and Psychoticism. All primary domains and the GSI were significantly increased in the PPA group compared to the CG group (Table 3).

**Table 3:** Self-reported psychiatric distress level in a sample of Swedish adolescent's according to psychosocial variable groups.

	**CG (*n* = 143)**	**PSP (*n* = 12)**	**PPA (*n* = 128)**	**Difference between groups**		
	***M (SD)*** **(Min–Max)**	***M (SD)*** **(Min–Max)**	***M (SD)*** **(Min–Max)**	**Test-stat (*H*)**	***p*-value**	***Post-hoc***
Somatization	0.62 (0.63) (0–3)	1.09 (0.87) (0–3)	0.97 (0.73) (0–3)	21.17	<0.001	CG < PPA^***^
Obsessive compulsive behavior	1.08 (0.81) (0–3)	1.42 (0.88) (0–3)	1.56 (0.86) (0–4)	21.59	<0.001	CG < PPA^***^
Psychoticism	0.36 (0.54) (0–2)	0.98 (0.92) (0–2)	0.78 (0.88) (0–4)	28.96	<0.001	CG < PPA^***^ CG < PSP^*^
Depression	0.77 (0.8) (0–4)	1.53 (1.11) (0–3)	1.35 (0.99) (0–4)	29.58	<0.001	CG < PPA^***^ CG < PSP^*^
Inetrpersonal sensitivity	0.7 (0.89) (0–4)	1.34 (1.01) (0–3)	1.29 (1.05AQ) (0–4)	29.06	<0.001	CG < PPA^**^
Hostility	0.66 (0.65) (0–3)	0.98 (0.72) (0.2)	1.12 (0.9) (0–4)	20.92	<0.001	CG < PPA^***^
Phobic anxiety	0.47 (0.72) (0–4)	0.85 (0.51) (0–2)	0.79 (0.91) (0–4)	17.54	<0.001	CG < PPA^*^
Anxiety	0.82 (0.73) (0–4)	1.58 (0.86) (0–3)	1.43 (0.9) (0–4)	38.39	<0.001	CG < PPA^***^ CG < PSP^*^
Paranoid ideation	0.71 (0.72) (0–3)	1.43 (1) (0–3)	1.39 (0.92) (0–4)	42.95	<0.001	CG < PPA^***^ CG < PSP^*^
GSI	0.72 (0.6) (0–3)	1.43 (0.69) (0–2)	1.17 (0.71) (0–4)	31.64	<0.001	CG < PPA^***^ CG < PSP^*^

*CG, comparison group; PSP, adolescents reporting Parental Substance use Problems; PPA, adolescents reporting the experience of Physical or Psychological Abuse*.

* *p < 0.05;*

** *p < 0.01;*

*** *p < 0.001*.

A gender specific-pattern was discovered when we made separate analyses of changes in female and male students' psychological distress level in association with the two negative psychosocial factors (PSP and PPA). Male students who reported having parents with substance use problems (PSP) had a significantly increased level of Phobic anxiety compared to CG and PPA groups (Table 4). The male students who reported the experienced of abuse (PPA) a significantly increased score in the domains of Psychoticism, Interpersonal sensitivity, Anxiety, and Paranoid ideation, as well as in their GSI was detected (Table 4).

**Table 4:** Self-reported psychiatric distress level in a sample of male adolescents according to psychosocial variable groups.

	**CG (*n* = 66)**	**PSP (*n* = 5)**	**PPA (*n* = 42)**	**Difference between groups**		
	***M (SD)*** **(Min–Max)**	***M (SD)*** **(Min–Max)**	***M (SD)*** **(Min–Max)**	**Test-stat (*H*)**	***p*-value**	***Post-hoc***
Somatization	0.41 (0.4) (0–2)	0.43 (0.31) (0–1)	0.58 (0.63) (0–3)	2.08	0.35	–
Obsessive compulsive behavior	0.77 (0.65) (0–3)	1.07 (0.89) (0–2)	1.13 (0.81) (0–3)	5.77	0.06	–
Psychoticism	0.18 (0.33) (0–1)	0.56 (1.04) (0–2)	0.54 (0.8) (0–4)	12.00	**0.002**	CG < PPA^*^
Depression	0.51 (0.67) (0–3)	0.9 (1.32) (0–3)	0.83 (0.84) (0–4)	4.91	0.09	–
Interpersonal sensitivity	0.35 (0.55) (0–2)	0.75 (1.13) (0–3)	0.68 (0.82) (0–4)	9.15	**0.010**	CG < PPA^**^
Hostility	0.57 (0.62) (0–3)	0.72 (0.56) (0–1)	0.96 (0.96) (0–4)	5.76	0.06	–
Phobic anxiety	0.16 (0.4) (0–2)	0.52 (0.36) (0–1)	0.31 (0.69) (0–4)	9.59	**0.008**	CG < PSP^**^ PPA < PSP^*^
Anxiety	0.43 (0.41) (0–2)	0.79 (0.83) (0–2)	0.85 (0.71) (0–4)	11.99	**0.002**	CG < PPA^**^
paranoid ideation	0.47 (0.59) (0–2.2)	1.61 (0.88) (0–3.6)	0.95 (0.83) (0–3.2)	12.07	**0.002**	CG < PPA^**^
GSI	0.44 (0.4) (0–2)	1.03 (0.99) (0–2)	0.80 (0.68) (0–4)	10.31	**0.006**	CG < PPA^**^

*CG, comparison group; PSP, adolescents reporting Parental Substance use Problems; PPA, adolescents reporting the experience of Physical or Psychological Abuse. M = Mean; SD = Standard Deviation*.

* *p < 0.05 and*

** *p < 0.01*.

A significant increased GSI and complaints in each of the nine primary domains were found in female students who reported one or several negative, psychosocial factor(s) in their life (Table 5). A distinct pattern could be recognized in female students who belong to the PSP group. Their psychological distress was significantly worse in the GSI and the primary domains of Paranoid ideation, Anxiety, Depression, and Psychoticism. Female students of the PPA group reported a significantly higher level of distress in each BSI domain compared to females of the CG group (Table 5).

**Table 5:** Self-reported psychiatric distress level in a sample of female adolescents according to psychosocial variable groups.

	**CG (*n* = 77)**	**PSP (*n* = 7)**	**PPA (*n* = 86)**	**Difference between groups**		
	***M (SD)*** **(Min–Max)**	***M (SD)*** **(Min–Max)**	***M (SD)*** **(Min–Max)**	**Test-stat (*H*)**	***p*-value**	***Post-hoc***
Somatization	0.78 (0.72) (0–3)	1.47 (0.87) (0–3)	1.16 (0.7) (0–3)	16.50	<0.001	CG < PPA^**^
Obsessive compulsive behavior	1.32 (0.84) (0–3)	1.77 (0.8) (1–3)	1.78 (0.81) (0–4)	12.98	0.002	CG < PPA^**^
Psychoticism	0.50 (0.63) (0–2)	1.29 (0.76) (0–2)	0.91 (0.9) (0–4)	16.72	<0.001	CG < PPA^**^ CG < PSP^*^
Depression	0.97 (0.84) (0–4)	1.98 (0.73) (1–3)	1.62 (0.96) (0–4)	25.36	<0.001	CG < PPA^***^ CG < PSP^**^
Interpersonal sensitivity	0.99 (1.00) (0–4)	1.83 (0.63) (1–3)	1.59 (1.02) (0–4)	18.06	<0.001	CG < PPA^***^
Hostility	0.73 (0.67) (0–3)	1.17 (0.79) (0–2)	1.2 (0.86) (0–3)	14.13	0.001	CG < PPA^**^
Phobic anxiety	0.71 (0.81) (0–4)	1.13 (0.47) (0–2)	1.03 (0.91) (0–4)	9.07	0.01	CG < PPA^*^
Anxiety	1.09 (0.79) (0–4)	2.02 (0.5) (1–3)	1.70 (0.85) (0–4)	26.60	<0.001	CG < PPA^***^ CG < PSP^**^
Paranoid ideation	0.90 (0.76) (0–2.8)	1.74 (0.72) (0.6–2.6)	1.61 (0.88) (0–3.6)	28.94	<0.001	CG < PPA^***^ CG < PSP^*^
GSI	0.91 (0.63) (0–3)	1.72 (1.44) (2–2)	1.36 (0.65) (0–3)	22.85	<0.001	CG < PPA^***^ CG < PSP^*^

*CG, comparison group; PSP, adolescents reporting Parental Substance use Problems; PPA, adolescents reporting the experience of Physical or Psychological Abuse*.

* *p < 0.05*,

** *p < 0.01, and*

*** *p < 0.001*.

### Somatic Complaints and Negative Psychosocial Factor

The most dramatic risk increase considering the prevalence of somatic complaints was found in the few participants who belong to the PSP group. For them the risk of having epilepsy increased 22 times (RR = 22.00), followed by approximately six times increased risk of having rheumatoid complaints (RR = 5.93) and gluten intolerance (RR = 5.50), and almost five times increased risk of constipation (RR = 4.64). Moreover, participants in the PSP group had a tripled risk to suffer from migraines (RR = 2.96). For those belonging to the PPA group, the risk to suffer from epilepsy (RR = 3.29), diarrhea (RR = 2.93), and constipation (RR = 2.92) were close to tripled, while the risk of having gluten intolerance and rheumatoid diseases (both values: RR = 2.21) was doubled (Table 6).

**Table 6:** Prevalence and risk ratio (RR) of defined somatic symptoms and diseases according to psychosocial groups.

	**CG (*n* = 143)**	**PSP (*****n*** **=** **12)**		**PPA (*****n*** **=** **128)**		**Chi-Square test**		
	**%**	**%**	**RR**	**%**	**RR**	**χ^**2**^**	***p*-value**	**Cramer's V**
Diarrhea	1.4	0.0	0.00	4.1	2.93	2.21	0.33	0.09
Constipation	3.6	16.7	4.64	10.5	2.92	4.28	0.12	0.13
Cancer	0.0	0.0	0.00	0.8	–	1.22	0.54	0.07
Epilepsy	0.7	15.4	22.00	2.3	3.29	4.59	0.10	0.13
Rheumatologic disease	1.4	8.3	5.93	3.1	2.21	0.56	0.75	0.05
Diabetes	1.4	0.0	0.00	0.0	0.00	1.99	0.37	0.09
Asthma	12.2	15.4	1.26	14.8	1.21	0.54	0.76	0.04
Allergy	30.2	41.7	1.38	30.4	1.00	0.66	0.72	0.05
Skin disease	7.4	0.0	0.00	6.3	0.85	0.99	0.61	0.06
Gluten intolerance	1.4	7.7	5.50	3.1	2.21	2.5	0.29	0.09
Migraine	13.0	38.5	2.96	19.5	1.50	7.00	0.03	0.16
Thyroid disease	0.7	0.0	0.00	0.8	1.14	0.08	0.96	0.02

*Significance level set at p < 0.017 after Bonferroni adjustment*.

*CG, comparison group; PSP, adolescents reporting Parental Substance use Problems; PPA, adolescents reporting the experience of Physical and/or Psychological Abuse*.

## Discussion

**Figure 4:**
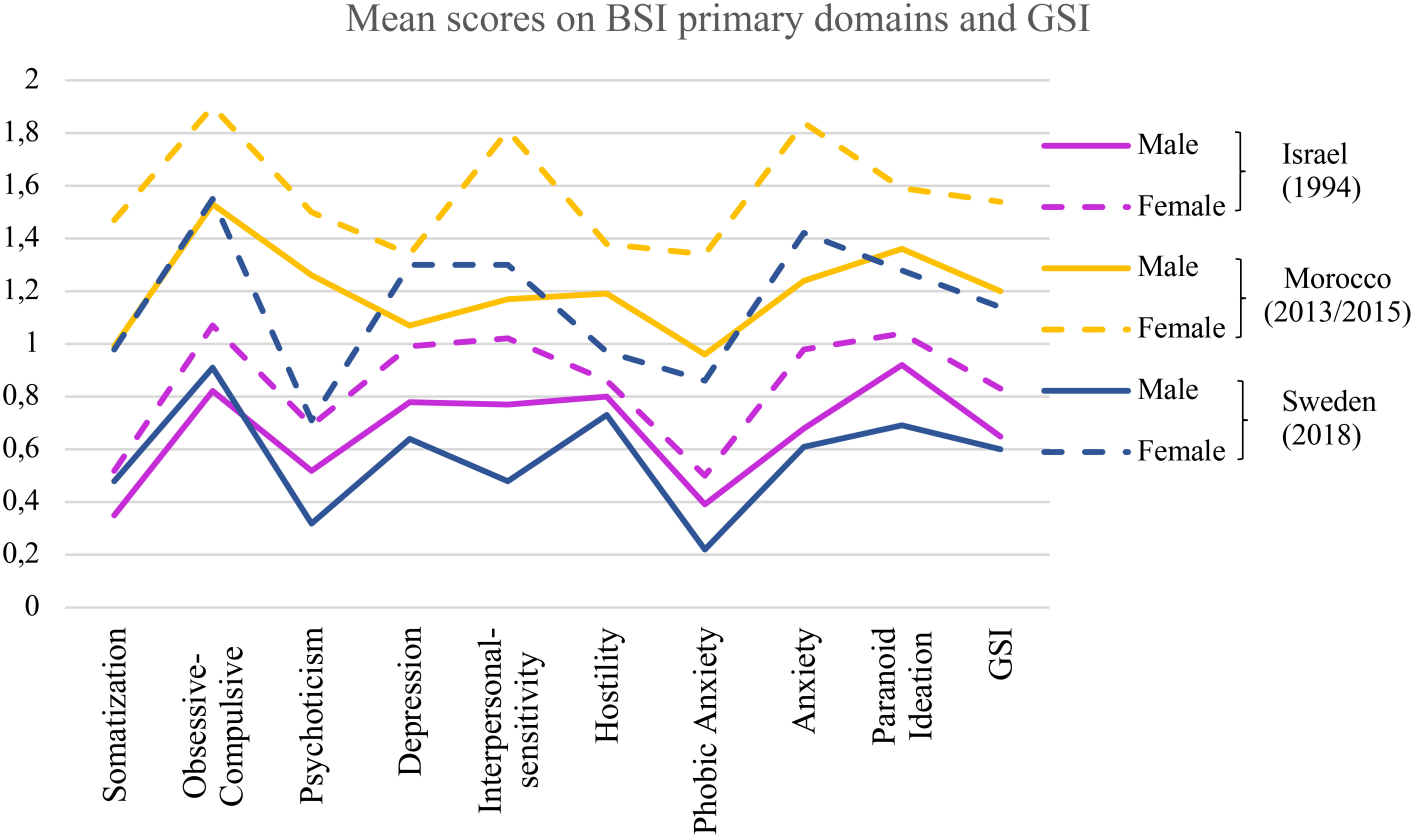
Psychological distress captured by BSI in the male and female adolescent populations of three countries' samples.

The measure of the General Severity Index (GSI) can be used as an overall indicator of mental health complaints. However, to day, only a few studies have investigated self-reported psychological complaints in a general population of high-school students (13, 42). When we compare GSI scores between those here referred studies, it becomes clear that Swedish high school students' general severity index (*M* = 0.93) is lower than that found in the Moroccan study (*M* = 1.38) (13), although our present Swedish results indicates higher general psychological distress than was found in an adolescent population of Israel (*M* = 0.83) (42) and America (*M* = 0.75) (43). However, a simple comparison of the studies' results in these four countries is not adequate, because the time span between the different data collections is rather long. Data in Sweden was collected in 2018, in Morocco 2013/2015, while the Israeli and American studies are from the beginning of the 1990s. Gender specific BSI data is available from the Isreali, Moroccan, and our study. To more fully comprehend the level of psychological distress captured by the BSI primary domaines, we visualized the three studies' gender specific findings (Figure 4). The level of psychological distress seems to sensitively reflect environmental changes in adolescents' life, and even with a few years of difference in the assessments specific differences between study results can be found (5).

The finding that female students experienced a significantly higher level of psychological distress than their male counterparts seems to be constant when we compare in time or between cultures. In the present study, Swedish female students compared to male students reported a higher level of psychological distress on each of the BSI primary domaines, a result in accordance with previous studies performed in other countries [e.g., GSI scores for male and female students were 0.65 and 0.83 in the Israeli study in 1994, 1.20 and 1.54 in the Moroccan study in 2014, and 0.6 and 1.14 in the present study, respectively; (5, 8, 9, 13, 42, 44–47)]. Domains that were found to most predominantly distinguish between gender by largest effect sizes were: “Anxiety” and “Interpersonal-sensitivity.” Anxiety as an important vulnerability factor in female adolescent development was previously discussed by Van Droogenbroeck et al. (5) in a study including Belgian adolescents and young adults. According to a WHO-based study including 42 countries (48), boys more often than girls have been found to have stress-related problems up to puberty, and the trend is reversed, i.e., at the age of 15, girls report more internalized emotional problems, which tend to last throughout most of the adulthood (8). Landstedt et al. (12) suggested that young females' anxiety and stress have their roots in experienced high demands in different areas of life, such as family and school. Other hypotheses about gender differences regarding stress-related problems suggest that girls' experience more physically drastic changes during puberty, more pronounced social demands, contradictory role expectations, and burn-out related to the school situation (49, 50).

An interesting detail of our study was that even hostility was rated higher by the female Swedish students (with a small effect size) compared to their male classmates. This finding has not previously been shown in adolescent populations in other countries. However, there are indications from previous studies that aggressive behavior (conduct disorder-like problem) is mostly influenced by specific environmental factors in girls (51). In this sense, our results may indicate the existence of new or stronger cultural and societal factors that may result in an increased distress in the form of hostility in female adolescents. Therefore, the higher level of hostility detected in females in the present study might be interpreted as a reaction to experienced threats to self-esteem or status and/or lack of respect. In this sense, hostility may grow out of anger (52). Our result may also imply the existence of an internalizing problem (53, 54), particularly the finding that the female adolescents also reported a higher level of anxiety. In fact, anxiety and pathological worry in general, are sometimes characterized by hostile traits, which may result in prompting the individual to adopt an attitude of closure toward the world and others after an episode of rage (55).

Questions that captured the highest distress level in the Swedish high school students were feeling nervous, being blocked in getting things done, doubts whether or not others can be trusted, difficulties in decision making, and getting easily hurt. The highest scores found on the BSI domain level were on the Obsessive compulsive behavior, Anxiety, Paranoid Ideation and Depression domains. The most commonly reported psychological health problems, according to several other studies summarizing young peoples' health, are generalized anxiety, panic syndrome, social anxiety, and depression (5, 8, 9). In a Moroccan, high-school population, the Obsessive compulsive behavior domain of BSI had the highest score, followed by Paranoid Ideation, Psychoticism, and Anxiety domains (13). Results from the Moroccan study suggest that high school students, in a different culture as Morocco, report quite similar psychological distress factors as the Swedish students in the present study; such as “trouble remembering things,” “problem getting things done,” “feeling a need to re-check things,” “being nervous,” “difficulties to trust others,” and “getting easily hurt.” An explanation to those similarities might be that there are common stress factors which are coupled to the intensive biopsychological changes during adolescence. In previous studies, students' self-related psychological, psychosomatic, and emotional problems were first and foremost associated with perceived stress over schoolwork and academic performance and conflicts with friends or family, or even with social media use (56), rather than perceived stress due to socioeconomic adversities (57–59).

### Somatic Complains

About 50% of the Swedish high school students reported one or several somatic complaints, a similar prevalence to that found in a large-scale nation-wide Swedish study including adolescents (60). There were no gender differences in the number of reported somatic complaints, a result that may contradict previous observations, where female adolescents reported more somatic complaints (6, 7, 60). The most prevalent somatic complaint in the present study was allergy (found in more than one-third of the study population), followed by migraine and asthma (reported by about 15%). Male students experienced more allergies, other than asthma, compared to female students, and this difference was significant. While migraine and constipation were significantly more often reported by female students. Gender-specific aspects of allergies have previously been coupled to immunological facts that point toward the differential role of sex hormones in immune functions (61). The most extensive study of allergy testing in the USA (62) showed that males have a higher, overall allergen sensitization rate than females of all ages. The higher prevalence of migraine and longer periods with constipation could follow explanations raised in the USA investigation, i.e., reasoning linked to the function of estrogen (63, 64). However, it could also easily be explained by the higher level of anxiety measured in our and others' studies (13, 65–67) and possibly by lower self-esteem (68) and higher academic demands and expectations in the female students (69), which also reflect the coexistence of mental and physical health problems.

### Negative Psychosocial Environmental Factors and Adolescent Mental and Somatic Health

There is obviously a need to reflect on the fact that “only” a tiny number (0.4%) of Swedish high-school students reported problems with parental alcohol use and only one male student admitted to a parental drug use problem. In recent years, nearly 6% of the Swedish population are estimated to be either dependent on alcohol or abuse alcohol (70). Based on the number of Swedish adults who are alcohol or drug-addicted, it seems rather strange that there are adolescents in the present study who do not recognize their parents' substance use as a problem. If Swedish students do not recognize parental substance use as a problem, it may lead to a lower threshold for them to start to use substances in their everyday life. It is well-known that broad social, economic and cultural factors are the key drivers of the prevalence of substance use. In fact, the norms of substance use vary considerably across cultures. The use of the same substance may be prohibited in one culture, tolerated in another, and even required in a third, meaning that the acceptance of substance and alcohol use depends on cultural norms (71). This can also be confirmed, if we compare the number of Swedish students recognizing parental substance use (including alcohol) as a problem with the number of Moroccan high school students reporting a parent with alcohol use problem (7, 11, 13). In Morocco, an Islamic culture, where substance use is prohibited and punished, 8.8% of the high school students indicated parental alcohol use as a problem. If a culture is prohibiting substance use, children might recognize their parents' substance use as a problem. While in cultures where the use of alcohol for example is incorporated as culinary arts, children do less frequently perceive their parents' alcohol use as problematic.

The strong cultural and societal impact on psychosocial environment in the youths' life could also be recognized in the prevalence of experienced physical or psychological abuse. An alarming number (47%) of the Swedish female adolescents—almost every second of them—and more than one third (37%) of the Swedish male adolescents reported the experience of some kind of abuse in their lives. Approximately 50% of them reported the experience of both physical and psychological abuse. In comparison, the prevalence of physical and psychological abuse reported by the female and male adolescents in the Moroccan study (7, 11, 13) were 17% and 13%, respectively. We hypothesize, that this significant discrepancy might be explained not only by cultural differences (between Sweden and Morocco) but also by a difference in time between the two data collections. In Sweden, data was collected in 2018, just following the “me-too” movement, which seemed to leave a substantial impact on a society that since long is influenced by a feministic movement. Data from Morocco was gathered between 2013 and 2015 (before the “me, too” movement swept through the world). This difference observed in percentage of reported abuse between the two populations may also be linked to the influence of different societal norms.

However, the impact of negative psychosocial factors on somatic and mental health can clearly be observed in both cultures (Swedish and Moroccan). The presence of any of the negative psychosocial factors was coupled with significantly worsened mental health (increased psychological distress) in our study, resembling the results found in the Moroccan population (13). The BSI domains that captured the increased psychological distress level with the presence of any negative psychosocial factor in the students' life were Psychoticism, Paranoid ideation, Anxiety, and Depression. The trauma of abuse or betrayal by parents, and living in a stressful, unsafe environment, may indeed lead to anxiety, depression, psychotic-like events, and increased suspiciousness, each based on fear and unpredictability of life events (72–75). In female students every aspect of psychological distress increased significantly in the presence of negative psychosocial factors, while in male students the scores of the Phobic anxiety domain increased most in those who reported parental substance use problems. The Psychoticism, Interpersonal-sensitivity, Paranoid ideation, and Anxiety domains' scores increased significantly in those male students who had experienced abuse. Based on these results it can be hypothesized that Swedish female adolescents in a given sociocultural environment do not respond unanimously with increased anxiety, suspicion, depression, interpersonal-sensitivity, and psychotic symptoms, but that a few of them may react with increased anger and hostility. Hardy et al. (76) studied gender differences in moral ideal self, the conceptualization of moral identity in relation to altruistic, aggressive, and norm-breaking behaviors of adolescents. They found that female adolescents had higher levels of moral ideal self than their male mates, and that moral ideal self was negatively correlated with aggression. It may appear that their results contraindicate our hypothesis. However, we argue that differences in the impact of cultures (Hardy and colleagues' study originating from US while our study is from Sweden where strong feministic ideologies in social, economic and political arenas are well-known components of the culture) and the differences in outcome measures (aggression and norm-breaking behavior vs. hostility) may allow both postulates. The presence of negative psychosocial factors in the present study did not significantly increase the presence of any of the somatic problems. However, the risk of having epilepsy, rheumatological problems, gluten intolerance (celiac), migraine, and constipation increased dramatically for participants who had negative psychosocial experiences.

As described previously negative environmental circumstances can change adolescent brain development on both a structural and functional level (77–79). Findings like that may contribute to—or explain—the increased prevalence of epilepsy in the studied group. As a consequence, or as a separate cascade, negative life events, such as being abused, might enhance biological susceptibility by an accelerated response to stressors, which augment the probability for inducing epileptic activity (80). Similarly, an increased risk for incidents of migraine could be explained by the neurological effects of abuse on brain functions (81) and amplified stress that can be involved in migraine pathogenesis (82).

The relationship between negative psychosocial factors and gastrointestinal problems is not a new finding. The experience of abuse is coupled to multi-component psycho-physiological consequences, which influence gastrointestinal reactivity, either directly or as a consequence of psychological comorbidities (83). This can occur via gut motility changes, especially as there is a variety of neural and humoral pathways that link the brain and the gut, which may be influenced by stress exposure (84). The exposure to stress alters the brain-gut interactions, leading to the development of a broad array of gastrointestinal disorders (85, 86). It is also important to recognize that gastrointestinal problems and neurological problems are closely linked to each other (87, 88).

The augmentation of the risk of having rheumatological problems in students who report negative psychosocial factors highlights the role of multiple stressors and increasing vulnerability to autoimmune disease. In a recent Swedish register study, including data from over 100,000 adult patients with stress-related disorders, 10 times as many matched unexposed individuals, and in more than 120,000 full siblings the association between stress-related disorders and autoimmune diseases were further proven, mainly in younger ages (89). Dube et al. (90) found in their study that individuals reporting two or more traumatic childhood events were at a 100% increased risk for rheumatic disease compared with those with no childhood trauma. The mechanisms presumed to underlie the associations between negative psychosocial factors and rheumatic problems include stress-related changes in the functioning of the autonomic, neuroendocrine, and/or immune systems (91).

## Strengths and Limitations

There are several limitations to be recognized in the present study. The MeSHe project has a cross-sectional design; consequently, no conclusions about causal associations can be drawn from the collected data. In addition, the data collection was limited to one high school in West Sweden and therefore, generalization of the results should be cautiously made. However, in that school the response rate was high (70%), which is a strength of our study. The high response rate is the result of the organized data collection during teacher supervised classes—while still ensuring anonymity to all respondent. Students with reading or writing disabilities, attention problems, and other complaints, were offered extra time and special pedagogue's help in the understanding of the survey. Therefore we can assume that the final study population was a representative sample. An obvious limitation of the study is that the data collection is based on self-report. Although, it has been shown that self-reported data are accurate when individuals understand the questions and when there is a strong sense of anonymity. Considering the existence of different diagnoses among respondents, it is suggested that self-report may be used as a proxy, when register data are unavailable (92). Importantly, the survey utilizes previously validated instruments for data assessment, and that is also a strength of our study.

## Conclusion

The results of the present study strengthen previous findings on gender differences in level of psychological distress and the significant impact of negative psychosocial factors on adolescents' physical and mental health. The study also presents new evidence about the decisive effect of culture and societal norms on the perception of threat and problem areas in the youths' life. The study concludes that the majority in the sample of Swedish high school students in 2018 reported none or very little psychological distress and none or only one somatic complaint. The most frequent psychological distress was coupled to obsession, anxiety, and the most frequent somatic complaint was allergy. Female students reported a higher level of psychological distress, but similar frequency of somatic complaints as their male classmates. The impact of parental substance use and the adolescents' experience of physical and/or psychological abuse seem to be substantial and a threat toward their mental health.

## Future Research

In future studies focusing on the enhancement of adolescent health and well-being we would suggest research approaches that are based on holistic methodology. The association between somatic and mental health and their susceptibility to the individual's unique and complex bio-psycho-social matrix is well-known and accepted today. However, health-, social care and educational systems are not yet adapted to this new and important knowledge. A significant, first step must be to make this comprehension available to all those who promote a life-long learning perspective and ensure that new academic programs are build on interdisciplinary grounds and holistic awareness.

## Data Availability Statement

The raw data supporting the conclusions of this article will be made available by the corresponding author upon request.

## Ethics Statement

The studies involving human participants were reviewed and approved by the Regional Ethical Review Board in Gothenburg; protocol No. 689–17. Written informed consent from the participants' legal guardian/next of kin was not required to participate in this study in accordance with the national legislation and the institutional requirements.

## Author Contributions

NK designed the study and grounded the MeSHe project, drafted the manuscript, supervised statistical calculations, and took responsibility for the intellectual content of the manuscript. BZ contributed to statistical analyses, took responsibility for the content of the results, and critically revised the manuscript. ST contributed to the manuscript with scientific responsibilities of references. SE contributed to the intellectual content and critically revision of the manuscript. All authors contributed to the article and approved the submitted version.

## Conflict of Interest

The authors declare that the research was conducted in the absence of any commercial or financial relationships that could be construed as a potential conflict of interest.

## Abbreviations

BSI: Brief Symptom Inventory
CG: comparison group
GSI: General Severity Index
M: mean
MeSHe: Mental and Somatic Health without borders
PPA: Physical and/or Psychological abuse
PSP: Parental Substance use Problems
RR: risk ratio
SD: standard deviation
SPSS: Statistical Package for the Social Sciences.
